# Association Rule Analysis for Validating Interrelationships of Combined Medication of Compound Kushen Injection in Treating Colon Carcinoma: A Hospital Information System-Based Real-World Study

**DOI:** 10.1155/2018/4579801

**Published:** 2018-08-30

**Authors:** Huisheng Yang, Yanming Xie, Jisheng Ni, Yue Liu, Rui Song, Cen Chen, Yan Zhuang, Yin Zhang

**Affiliations:** ^1^Institute of Basic Research in Clinical Medicine, China Academy of Chinese Medical Sciences, Dongcheng District, Beijing 100700, China; ^2^Institute of Acupuncture and Moxibustion, China Academy of Chinese Medical Sciences, Dongcheng District, Beijing 100700, China; ^3^Southeast University-Monash University Joint Research Institute, Wuzhong District, Southeast University, Suzhou 215123, China; ^4^School of Chinese Medicine, Beijing University of Chinese Medicine, Chaoyang District, Beijing 100029, China; ^5^Longwood Translation Medicine China Initiative, Boston, MA 02115, USA; ^6^School of Statistics, Renmin University of China, Haidian District, Beijing 100872, China; ^7^Center of Computer Management, Navy General Hospital of PLA, Haidian District, Beijing 100048, China

## Abstract

**Background:**

Real world evidence is important for informing healthcare practice and developing medical products and has gained broad interest in healthcare. Compound Kushen Injection (CKI) has been widely applied into treatment of colon carcinoma (CC) in China. Postapproval drug assessment related retrospective studies using electrical medical record (EMR) collected from hospital information system (HIS) is one of the most important categories of real-world study (RWS). Based on HIS EMR, interrelationships of combined medication of CKI in treating CC can be validated in real world settings.

**Methods:**

This study was conducted based on a large-scale integrated database of EMR derived from HIS. EMR of 3328 patients initially diagnosed with CC among 49,597 patients treated with CKI were included in the study. Descriptive statistical analyses and apriori algorithm based association rule analyses were performed, respectively, to validate frequency distribution and interrelationships of combined medication of CKI in treating CC.

**Results:**

The pharmacological mechanisms of TCMs that have been commonly used in conjunction with CKI include heat-clearing and detoxifying, qi-reinforcing, blood circulation-promoting and stasis-removing, blood-stanching, and qi-regulating. For modern medicines, antibiotics, antineoplastic chemotherapeutic drugs, immunomodulator, 5-HT receptor antagonist drugs, and corticosteroids are most often combined with CKI. The association rules of medication combinations of CKI in treating CC in real world manifest certain laws for both TCMs and modern medicines. They are generally in line with CC treatment guidelines.

**Conclusions:**

It is a common practice for CKI to be integrated with both modern medicines and TCMs when treating CC in China. The associations of medication combinations of CKI in treating CC manifest certain laws for both TCMs and modern medicines. The RWS for validating interrelationships of combined medication may provide evidence for rational use of CKI. Further explorations are needed to verify and expand the conclusions.

## 1. Background

Compound Kushen Injection (CKI), is a pure TCM extracted from two herbs,* Radix Sophorae Flavescentis* and* Rhizoma Hterosmilacis*. In TCM theory, CKI has the effect of clearing heat, promoting diuresis, removing pathogenic and toxic material from the body [1]. With the effective components of oxymatrine, oxysophocarpine, and matrine [2], it has been widely applied into treatment of various kinds of malignant tumors in China, including colon carcinoma (CC) [3]. CKI has been listed in the Drug Directory for National Medical Insurance, Employment Injury Insurance, and Maternity Insurance [4]. It is also listed as therapeutic medication for CC according to Guideline for Diagnosis and Treatment of Tumor in TCM [5] published by China Association of Chinese Medicine in 2008 and Clinical Practice Guidelines of Chinese Medicine in Oncology [6] issued by China Academy of Chinese Medical Sciences in 2014.

Trails have showed that CKI can improve overall efficiency of the treatment for multiple malignant tumors, relieve clinical symptoms such as cancer pain, fever, and fatigue, and potentiate the efficacy of chemotherapy and radiotherapy for CC with additional toxicity reduction effect [3, 7, 8]. The mechanisms of CKI comprise inhibiting the proliferation and metastasis of tumor cells [9–11], inducing the differentiation and apoptosis of tumor cells [12, 13], restraining the formation of tumor neovascularization [14], suppressing tumors' drug resistance [15], and inducing the autophagy of tumor cells [16, 17]. Previous studies show that compared with pure chemotherapy treatment, CKI combined with chemotherapy can improve clinical effects and patients' life quality, extend lifetime, and reduce the toxicity of chemotherapy [18–20]. The mechanism of which includes improving the immunity of patients with CC [21], restraining the proliferation of colon cancer cells and inducing their apoptosis [22, 23], suppressing the formation of tumor neovascularization [24], and curbing the activation of NF-*κ*B inside macrophage [25].

Real-world studies (RWS) include a spectrum of studies that apply various methods to data collected from real world settings [26]. Real world evidence is important for informing healthcare practice and developing medical products and has gained broad interest in healthcare [27]. In China, the term “real world evidence” was not explicitly used until 2010, when researchers from our group in Institute of Basic Research in Clinical Medicine (IBRCM), China Academy of Traditional Chinese Medical Sciences (CACMS), carried out the first RWS to evaluate traditional Chinese medicine interventions [28]. Retrospective studies using electrical medical record (EMR) collected from hospital information system (HIS) are one of the most important categories of RWS [27] and are important for postapproval drug assessment [29], healthcare quality improvement [30], and new indications of medical products [31].

EMR stored in HIS have inherent strengths of high reliability of sources, large scale of samples, accuracy of recording, reasonable framework, and abundance in dimensions. Particularly, it records detailed medication orders throughout the whole treatment process at the time of hospitalization [32]. The laws of combined medication can thus be found through the large quantity of data provided by HIS. Considering CKI has been widely applied into treatment of CC in China, our present study aimed to validate the interrelationships of combined medication of CKI in the treatment of CC by using HIS EMR and thus provide evidence for rational use of CKI in real world settings.

## 2. Methods

### 2.1. Data Sources

This study was conducted based on a large-scale integrated data warehouse of EMR from HIS of 39 Class A tertiary hospitals in China, that was built by IBRCM of CACMS [26, 33, 34]. EMR of patients whose first-listed diagnoses were CC and were treated with CKI were extracted from 22 hospitals among the above-mentioned medical centers.

### 2.2. Standardization of Database Structure

Due to the difference in data structure of HIS of varied hospitals, IBRCM, by standardizing original data structure, built an integrated database with the same structure of variables that contained general information, diagnosis information, medication orders, and laboratory test results. Patient's ID is the only index that links different data subsets.

### 2.3. Data Standardization

All analyses were made on account of standardized modern medicine diagnosis information and medication orders. Disease names were standardized with reference to ICD-10 [35]. Chinese patent medicines with the same ingredients but in different drug forms were standardized and merged, while their TCM theory based pharmacological mechanisms were classified in accordance with their major functions. Modern medicines were standardized by translating their trade name into chemical name (if applicable), and their pharmacological effects were normalized and categorized with reference to* Pharmacopoeia of the People's Republic of China* (2010) [36].

### 2.4. Exclusion Criteria

Exclusion criteria of combined medicines are as follows: (1) solvents, including glucose injection, sodium chloride injection, and glucose and sodium chloride injection, were excluded; (2) potassium chloride and vitamins (except for Vitamin C) were excluded; (3) insulin when combined with glucose injection or glucose and sodium chloride injection was excluded; (4) heparin only when administrated through intravenous drip, intravenous injection, pumping, or subcutaneous injection was excluded; (5) combined drugs the medication administration time of which did not fall into that of CKI were excluded.

### 2.5. Data Analysis

Descriptive statistical analyses in this study were carried out using SAS software* (version 9.3, SAS Institute Inc., Cary, NC, U.S.A). *Considering the complexity of drug combination, only the medicines that have been frequently used in conjunction with CKI (top 20 excerpted) were included for data mining analyses. Apriori algorithm based association rule analysis (ARA) and plotting in this study were processed by SPSS Clementine software* (version 12.0, SPSS Inc., Chicago, IL, U.S.A)*.

## 3. Results

### 3.1. Characteristics of General Information

EMR of 3328 patients first diagnosed with CC among 49597 patients who were treated with CKI at the time of hospitalization were included in the study. The earliest record of case was August 2002, while the latest one was December 2014. The characteristics are as follows: gender: male: 1953 cases, female: 1353 cases, and missing: 22 cases; age: 61.85±13.68 years old; length of hospital stays: 11.96±6.43 days; course of CKI treatment: 9.32±6.28 days; single dosage of CKI: 16.73±5.63 ml; daily dosage of CKI: 15.84±4.17 ml.

### 3.2. Distribution Characteristics of Combined TCMs

227 traditional Chinese medicines were used in conjunction with CKI. Top 20 were tabulated based on the frequency of use (Table 1).

### 3.3. Distribution Characteristics of Combined Modern Medicines

760 modern medicines were used in conjunction with CKI. Top 20 were tabulated based on the frequency of use (Table 2).

### 3.4. TCM Pharmacological Mechanism Distribution Characteristics of Combined TCMs

Frequency order of pharmacological mechanism of combined TCMs (top 20) is shown in Table 3.

### 3.5. Pharmacological Mechanism Distribution Characteristics of Combined Modern Medicines

Frequency order of pharmacological mechanism of combined modern medicines (top 20) is shown in Table 4.

### 3.6. ARA of Combined TCMs

**Figure 1:**
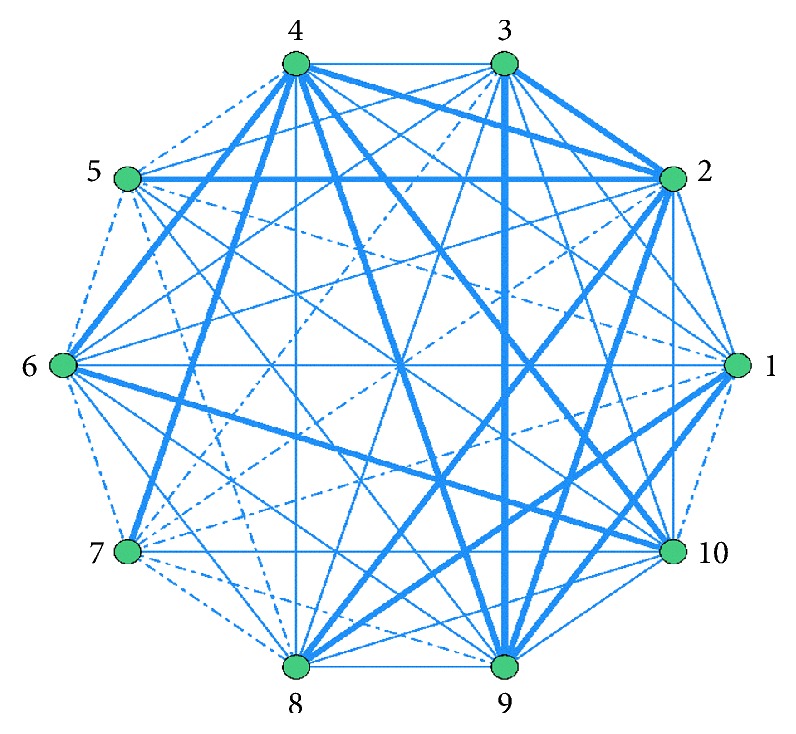
Network diagram of association rules of TCM combined with CKI. (1) Shenqi Fuzheng injection; (2) Yadanzi Youru injection; (3) Aidi injection; (4) Ganmao Qingre granules; (5) Kangai injection; (6) Simotang oral liquid; (7) Jianpi Yishen granules; (8) Xiaoaiping injection; (9) Yunnan Baiyao capsules; (10) Zhenqi Fuzheng granules.

TCMs are used in conjunction with CKI. The association rules between different medicines obtained by ARA are ordered by Support. Top 10 are listed in Table 5. The features are visually presented based on network of associations in Figure 1.

In Figure 1, in order to show the difference of correlation between combined drugs, use frequency≧1.06% is represented by bold line; use frequency≦0.5% is represented by dotted line; use frequency between 0.5% and 1.06% is represented by fine line.

### 3.7. ARA of Combined Modern Medicines and Merged Analysis

**Figure 2:**
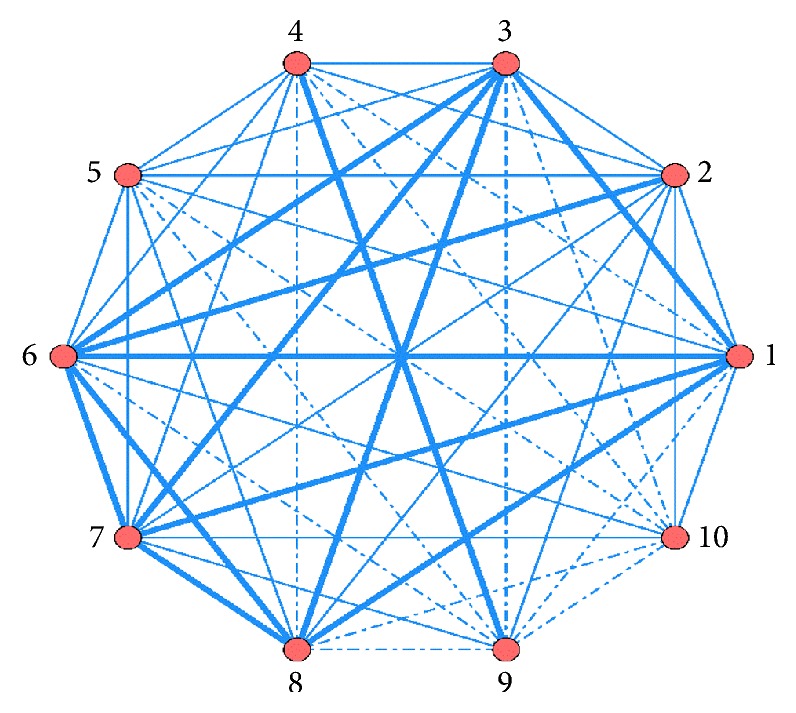
Network diagram of association rules of modern medicines combined with CKI. (1) Oxaliplatin; (2) Dexamethasone; (3) Fluorouracil; (4) Amino acid; (5) Metoclopramide; (6) Tropisetron; (7) Thymosin; (8) Leucovorin; (9) Medium- and long-chain fat emulsion; (10) Pantoprazole sodium.

**Figure 3:**
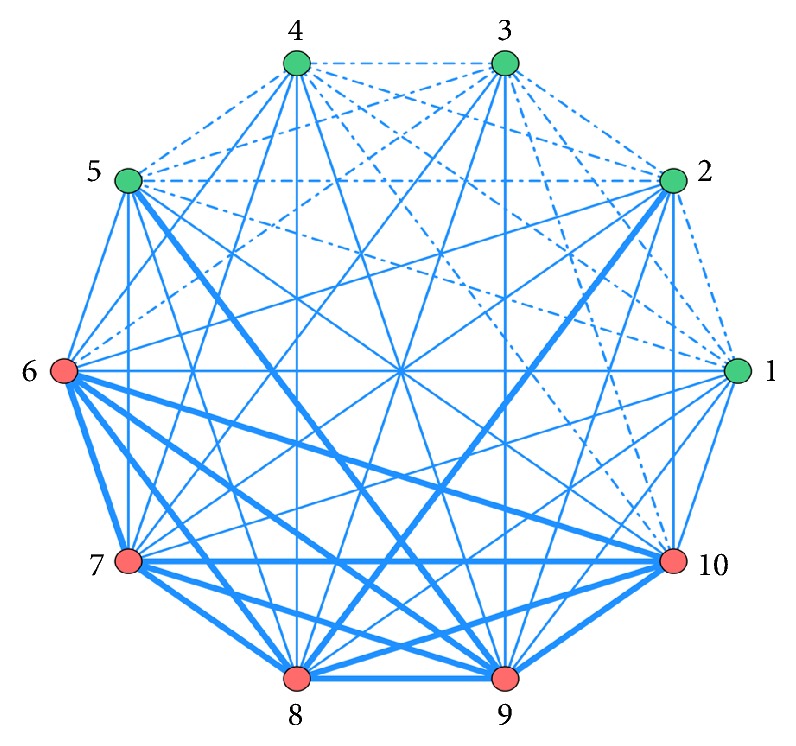
Network diagram of association rules of CKI combined with both TCM and modern medicines. (1) Shenqi Fuzheng injection; (2) Yadanzi Youru injection; (3) Aidi injection; (4) Ganmao Qingre granules; (5) Zhenqi Fuzheng granules; (6) Oxaliplatin; (7) Fluorouracil; (8) Tropisetron; (9) Thymosin; (10) Leucovorin.

Modern medicines are used in conjunction with CKI. The association rules between different medicines obtained by ARA are ordered by Support. Top 10 are listed in Table 6. The features are visually presented based on network of associations in Figure 2. In merged analysis, the network of associations is shown in Figure 3.

In Figure 2, use frequency≧20.3% is represented by bold line; use frequency≦12% is represented by dotted line; use frequency between 12% and 20.3% is represented by fine line.

In Figure 3, use frequency≧7.49% is represented by bold line; use frequency≦2.87% is represented by dotted line; use frequency between 2.87% and 7.49% is represented by fine line.

### 3.8. ARA of Pharmacological Mechanisms of Combined TCMs

**Figure 4:**
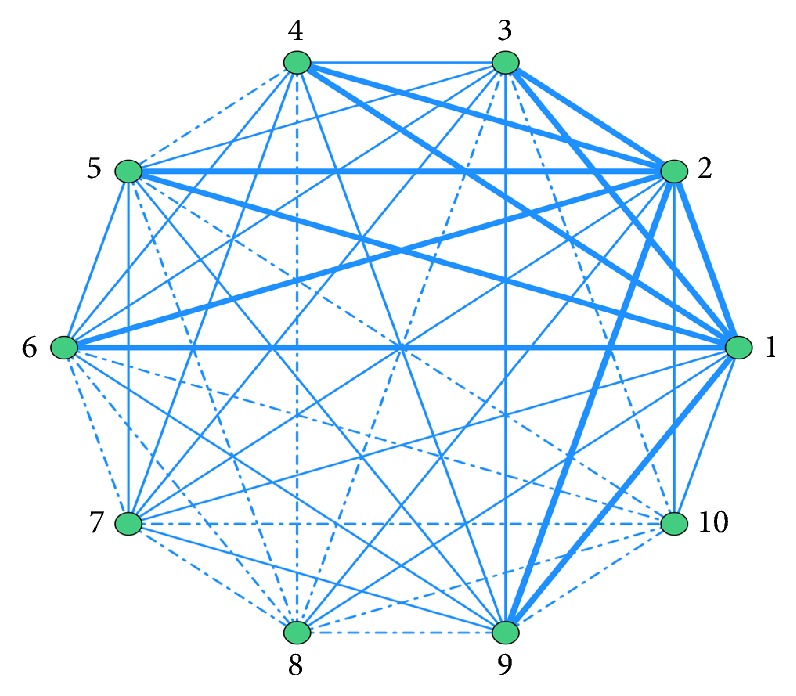
Network diagram of pharmacological mechanism association rules of TCMs combined with CKI. (1) Qi-reinforcing; (2) heat-clearing and detoxifying; (3) blood circulation-promoting and stasis-removing; (4) spleen-invigorating and stomach-harmonizing; (5) Qi-regulating; (6) bowel-relaxing; (7) blood-regulating; (8) Blood stasis-removing; (9) blood-stanching; (10) swelling-reducing and mass-resolving.

The association rules between different pharmacological mechanisms of combined TCMs obtained by ARA are ordered by Support. Top 10 are listed in Table 7. The features are visually presented based on network of associations in Figure 4.

In Figure 4, in order to show the difference of correlation of pharmacological mechanism between combined drugs, use frequency≧2.08% is represented by bold line; use frequency≦0.42% is represented by dotted line; use frequency between 0.42% and 2.08% is represented by fine line.

### 3.9. ARA of Pharmacological Mechanism of Combined Modern Medicines, and Merged Analysis

**Figure 5:**
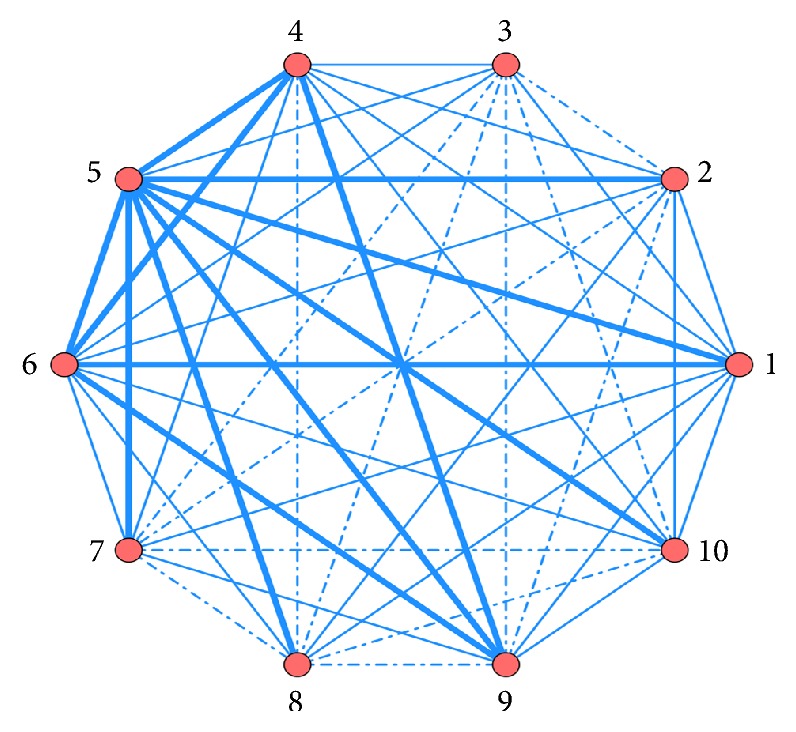
Network diagram of pharmacological mechanism association rules of modern medicines combined with CKI. (1) 5-HT receptor antagonist; (2) hepatic protector; (3) dopamine receptor antagonist; (4) antibiotics; (5) antineoplastic chemotherapeutic drugs; (6) immunomodulator; (7) corticosteroids; (8) antifolate; (9) nutritious drugs; (10) proton pump inhibitor.

**Figure 6:**
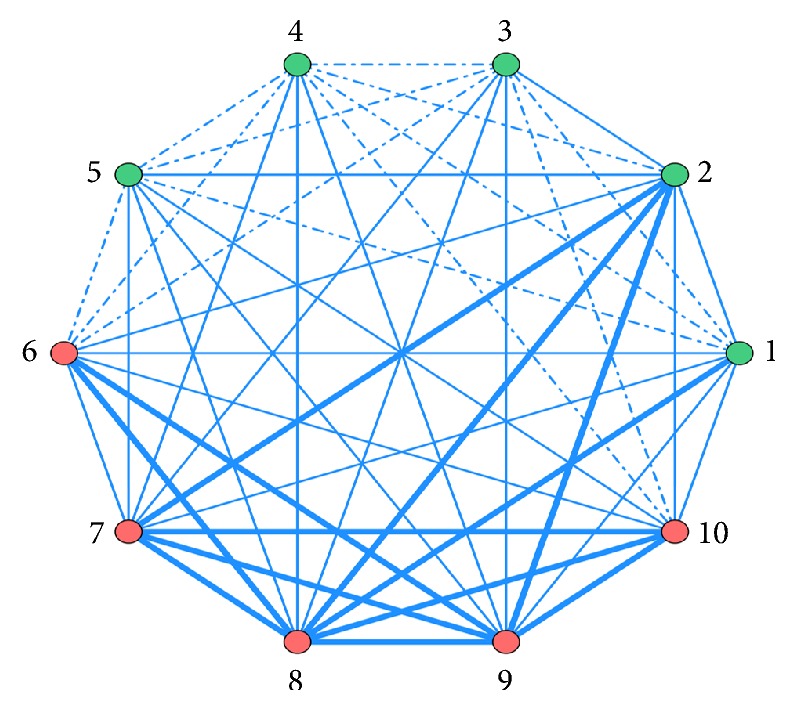
Network diagram of pharmacological mechanism association rules of CKI combined with both TCM and modern medicines. (1) Qi-reinforcing; (2) heat-clearing and detoxifying; (3) blood circulation-promoting and stasis-removing; (4) Qi-regulating; (5) blood-stanching; (6) 5-HT receptor antagonist drugs; (7) antibiotics; (8) antineoplastic chemotherapeutic drugs; (9) immunomodulator; (10) nutritious drugs.

Modern medicines are used in conjunction with CKI. The association rules between different pharmacological mechanisms of combined modern medicines obtained by ARA are ordered by Support. Top 10 are listed in Table 8. The features are visually presented based on network of associations in Figure 5. In merged analysis, the network of associations is shown in Figure 6.

In Figure 5, use frequency≧30.4% is represented by bold line; use frequency≦17.4% is represented by dotted line; use frequency between 17.4% and 30.4% is represented by fine line.

In Figure 6, use frequency≧28.1% is represented by bold line; use frequency≦5.15% is represented by dotted line; use frequency between 5.15% and 28.1% is represented by fine line.

## 4. Discussion

ARA is widely used to analyze internal connections hidden in item sets of multidimensional data [37–39]. In this study, ARA is performed to generate candidate item sets under a threshold control of support and confidence and finally identify association rules that highlight general trends in the database of combined TCMs and modern medicines. Association rules are presented in the implicative expression of A => B. Support (A -> B) = P (A U B). Support equals the probability of coadministration of drugs A and B. It is used to assess the frequency and importance of association rules. Confidence (A -> B) = P (A∣B). Confidence equals the probability of administration of drug B after drug A is used. It is capable of assessing the intensity and reliability of association rules [40].

In terms of features of combination with other TCMs, CKI is most often administrated in conjunction with TCMs with the pharmacological mechanisms of qi-reinforcing, heat-clearing and detoxifying, blood circulation-promoting and stasis-removing, spleen-invigorating and stomach-harmonizing, and qi-regulating. The common combinations include the following: (1) on the basis of combination of CKI and qi-reinforcing, using one of the following: heat-clearing and detoxifying, blood circulation-promoting and stasis-removing, blood-stanching, bowel-relaxing, qi-regulating, spleen-invigorating and stomach-harmonizing, and blood-regulating; (2) on the basis of combination of CKI and heat-clearing and detoxifying, using one of the following: blood circulation-promoting and stasis-removing, blood-stanching, bowel-relaxing, qi-regulating, spleen-invigorating and stomach-harmonizing, blood-regulating, swelling-reducing and mass-resolving, for yin-tonifying, for reviving yang to save from collapse, qi-reinforcing and blood-nourishing; (3) on the basis of combination of CKI and blood circulation-promoting and stasis-removing, using blood-stanching and qi-regulating; (4) on the basis of combination of CKI and qi-regulating, using bowel-relaxing used. In TCM theory, a number of pathogenic factors cause the malfunction of large intestine and stagnant movement of qi, blood, and body fluid, leading to certain pathological changes such as stagnation of qi and blood, phlegm stasis, damp turbidity, and heat-toxicity. Stagnated in large intestine, these pathological products interact with each other and eventually form tangible lumps as time goes by. The above combinations of TCMs when treating CC have effects of reinforcing healthy qi, clearing heat and detoxication, reinforcing qi and invigorating spleen, eliminating dampness and regulating the stomach, regulating qi and relieving pain, smoothing qi, and removing stasis. They are in line with* Guideline for Diagnosis and Treatment of Tumor in TCM *published by China Association of Chinese Medicine [5].

In terms of features of combination with modern medicines, CKI is most often administrated in conjunction with antibiotics, antineoplastic chemotherapeutic drugs, immunomodulator, 5-HT receptor antagonist drugs, and corticosteroids. The common combinations include the following: (1) on the basis of combination of CKI and antineoplastic chemotherapeutic drugs, using one of the following drugs: immunomodulator, 5-HT receptor antagonist drugs, antibiotics, antifolate, nutritious drugs, corticosteroids, hepatic protector, proton pump inhibitor, dopamine receptor antagonist; (2) on the basis of combination of CKI and immunomodulator, using one of the following drugs: 5-HT receptor antagonist drugs, antibiotics, nutritious drugs, hepatic protector, proton pump inhibitor; (3) on the basis of combination of CKI and antibiotics, using nutritious drugs; (4) on the basis of combination of CKI and corticosteroids, using antifolate, dopamine receptor antagonist, and antineoplastic drugs; (5) CKI being administrated in conjunction with antibiotics, antineoplastic chemotherapeutic drugs, immunomodulator, 5-HT receptor antagonist drugs, and corticosteroids. According to guidelines [41–43], major therapeutic strategy to treat CC includes chemotherapy before operation and administration of antibiotics, immunomodulator, and corticosteroids after operation. Antineoplastic chemotherapeutic drugs, antibiotics, and immunomodulator are strongly recommended with a view to raising total survival rate, preventing postoperative infection, prolonging survival period for recurrent patients, and improving life quality. The above combinations have effects of inhibiting the proliferation of CC cells, preventing infection, alleviating the side effect of radiotherapy and chemotherapy, and mitigating local compression and edema. They are confronted with clinical guidelines for diagnosis and treatment of CC [42, 44, 45].

In merged analysis, the common combinations include the following: (1) on the basis of combination of CKI and heat-clearing and detoxifying, antineoplastic chemotherapeutic drugs and immunomodulator are used at the same time; (2) on the basis of combination of CKI and qi-reinforcing, antineoplastic chemotherapeutic drugs are used; (3) on the basis of combination of CKI and antibiotics, antineoplastic chemotherapeutic drugs, and immunomodulator are used at the same time; (4) on the basis of combination of CKI and antineoplastic chemotherapeutic drugs, 5-HT receptor antagonist drugs, and corticosteroids are used, respectively; (5) on the basis of combination of CKI and 5-HT receptor antagonist drugs, corticosteroids are used; (6) on the basis of combination of CKI and immunomodulator, either antineoplastic chemotherapeutic drugs, 5-HT receptor antagonist drugs, or corticosteroids is added. The combination of TCM and chemotherapeutics has been proved to have the effect of relieving symptoms, raising life quality, strengthening immune functions, and alleviating the side effect of chemotherapy when treating CC [18, 46, 47].

Strengths of our present study should be noted. (1) The data source of this study is of high quality. The large-scale integrated data warehouse records EMR of over three million cases from HIS of 39 Class A tertiary hospitals nationwide in China. It covers demographic data, diagnosis information of TCM and modern medicine, medication orders, common clinical test results, and treatment outcomes [33, 34]. (2) Standardization of database structure, standardization of different categories of variables, and strict logic checking were performed before analysis to ensure quality control. (3) The advantages of ARA include good adaptability for analysis of multidimensional and nonlinear medication and disease related variables [48].

Disadvantages of this study should also be addressed. (1) HIS EMR is derived from real-world records in the process of clinical treatment and is not originally designed for research purposes. (2) Selection bias may exist because data were derived from participants in 22 hospitals in China, and therefore the cases are likely not representative of patients in other medical centers nationwide. (3) Apriori algorithm generates a large quantity of candidate sets in the ARA procedure by repeatedly scanning all the records in database. Hence, such large amount of calculation by apriori algorithm may consume too many resources when it comes to the analysis of large-scale database.

## 5. Conclusion

CKI has been used extensively integrated with both modern medicines and TCMs when treating CC in China. The pharmacological mechanisms of TCMs that most frequently combined with CKI include heat-clearing and detoxifying, qi-reinforcing, blood circulation-promoting and stasis-removing, blood-stanching, and qi-regulating. For modern medicines, antibiotics, antineoplastic chemotherapeutic drugs, immunomodulator, 5-HT receptor antagonist drugs, and corticosteroids are most often combined with CKI. The associations of medication combinations of CKI in treating CC in real world manifest certain laws for both TCMs and modern medicines. Further explorations are needed to verify and expand the conclusions.

## Figures and Tables

**Table 1:** Frequency of TCMs combined with CKI (Top 20, N=3328).

Frequency sequence	Medication	Distribution frequency (%)	Frequency sequence	Medication	Distribution frequency (%)
1	Yadanzi Youru injection	443 (13.31)	11	Lianqi capsules	110 (3.31)
2	Zhenqi Fuzheng granules	345 (10.37)	12	Kangfuxin liquid	109 (3.28)
3	Shenqi Fuzheng injection	317 (9.53)	13	Shengmai injection	91 (2.73)
4	Aidi injection	297 (8.92)	14	Tongbianling capsules	84 (2.52)
5	Ganmao Qingre granules	295 (8.86)	15	Qirong Runchang oral liquid	78 (2.34)
6	Yunnan Baiyao capsules	261 (7.84)	16	Tanreqing injection	77 (2.31)
7	Simotang oral liquid	214 (6.43)	17	Shengmai II oral liquid	74 (2.22)
8	Kangai injection	182 (5.47)	18	Shenfu injection	73 (2.19)
9	Xiaoaiping injection	170 (5.11)	19	Qingkailing injection	71 (2.13)
10	Jianpi Yishen granules	138 (4.15)	20	Xihuang capsules	69 (2.07)

**Table 2:** Frequency of modern medicines combined with CKI (Top 20, N=3328).

Frequency sequence	Medication	Distribution frequency (%)	Frequency sequence	Medication	Distribution frequency (%)
1	Tropisetron	1599 (48.05)	11	Ornidazole	660 (19.83)
2	Thymosin	1554 (46.69)	12	Lidocaine	651 (19.56)
3	Oxaliplatin	1509 (45.34)	13	Recombinant human Interleukin 2	636 (19.11)
4	Fluorouracil	1361 (40.90)	14	Reduced glutathione	629 (18.90)
5	Leucovorin	1271 (38.19)	15	Alanyl-glutamine	623 (18.72)
6	Dexamethasone	1250 (37.56)	16	Furosemide	590 (17.73)
7	Metoclopramide	1018 (30.59)	17	Cinobufagin	569 (17.10)
8	Amino acid	964 (28.97)	18	Ambroxol	550 (16.53)
9	Medium- and long-chain fat emulsion	794 (23.86)	19	Omeprazole	550 (16.53)
10	Pantoprazole sodium	794 (23.86)	20	Human serum albumin	527 (15.84)

**Table 3:** Frequency of TCM pharmacological mechanisms of TCMs combined with CKI (Top 20, N=3328).

Frequency sequence	TCM Pharmacological mechanism	Distribution frequency (%)	Frequency sequence	TCM Pharmacological mechanism	Distribution frequency (%)
1	Heat-clearing and detoxifying	1535 (46.12)	11	Reviving yang to save from collapse	73 (2.19)
2	Qi-reinforcing	1159 (34.83)	12	Qi-reinforcing and blood-nourishing	52 (1.56)
3	Blood circulation-promoting and stasis-removing	312 (9.38)	13	Warming interior	49 (1.47)
4	Blood-stanching	294 (8.83)	14	Promoting circulation of qi and blood to relieve pain	49 (1.47)
5	Qi-regulating	273 (8.20)	15	Cough-relieving	39 (1.17)
6	Bowel-relaxing	260 (7.81)	16	Cough-preventing	33 (0.99)
7	Spleen-invigorating and stomach-harmonizing	163 (4.90)	17	Digestion-promoting	32 (0.96)
8	Swelling-reducing and mass-resolving	103 (3.09)	18	Phlegm-eliminating	31 (0.93)
9	Blood-regulating	94 (2.82)	19	Blood-nourishing and tranquilization	25 (0.75)
10	Yin-tonifying	75 (2.25)	20	Yang-tonifying	23 (0.69)

**Table 4:** Frequency of pharmacological mechanisms of modern medicines combined with CKI (Top 20, N=3328).

Frequency sequence	Pharmacological mechanism	Distribution frequency (%)	Frequency sequence	Pharmacological mechanism	Distribution frequency (%)
1	Antineoplastic chemotherapeutic drugs	2760 (82.93)	11	Painkiller	1030 (30.95)
2	Immunomodulator	2308 (69.35)	12	Tranquilizer	947 (28.46)
3	Antibiotics	1676 (50.36)	13	Medicine for electrolyte balance adjustment	851 (25.57)
4	5-HT receptor antagonist drugs	1658 (49.82)	14	Anesthetic	799 (24.01)
5	Nutritious drugs	1386 (41.65)	15	Diuretics	786 (23.62)
6	Hepatic protector	1303 (39.15)	16	H2 receptor antagonist drugs	755 (22.69)
7	Proton pump inhibitor	1243 (37.35)	17	Analgesic and anti-inflammatory drugs	668 (20.07)
8	Antifolate	1211 (36.39)	18	Cell differentiation drugs	607 (18.24)
9	Corticosteroids	1152 (34.62)	19	Antianemics	603 (18.12)
10	Dopamine receptor antagonist	1063 (31.94)	20	Expectorant	557 (16.74)

**Table 5:** Association rules of TCM combined with CKI.

No.	Association rules	Support	Confidence
1	Zhenqi Fuzheng granules =>Ganmao Qingre granules	1.472	14.2
2	Ganmao Qingre granules =>Zhenqi Fuzheng granules	1.472	16.6
3	Aidi injection =>Yunnan Baiyao capsules	1.322	14.8
4	Yunnan Baiyao capsules =>Aidi injection	1.322	16.9
5	Aidi injection =>Yadanzi Youru injection	1.322	14.8
6	Yunnan Baiyao capsules =>Shenqi Fuzheng injection	1.292	16.5
7	Ganmao Qingre granules =>Yadanzi Youru injection	1.292	14.6
8	Simotang oral liquid =>Ganmao Qingre granules	1.262	19.6
9	Ganmao Qingre granules =>Simotang oral liquid	1.262	14.2
10	Yunnan Baiyao capsules =>Yadanzi Youru injection	1.262	16.1

**Table 6:** Association rules of modern medicines combined with CKI.

No.	Association rules	Support	Confidence
1	Leucovorin =>Oxaliplatin	33.4	87.3
2	Oxaliplatin =>Leucovorin	33.4	73.6
3	Oxaliplatin =>Tropisetron	30.3	66.8
4	Tropisetron =>Oxaliplatin	30.3	63.0
5	Leucovorin =>Fluorouracil	28.9	75.6
6	Fluorouracil =>Leucovorin	28.9	70.6
7	Fluorouracil =>Tropisetron	28.2	68.9
8	Tropisetron =>Fluorouracil	28.2	58.7
9	Leucovorin =>Tropisetron	27.7	72.5
10	Tropisetron =>Leucovorin	27.7	57.6

**Table 7:** Association rules of pharmacological mechanism of TCMs combined with CKI.

No.	Association rules	Support	Confidence
1	Qi-reinforcing =>Heat-clearing and detoxifying	16.23	46.6
2	Heat-clearing and detoxifying =>Qi-reinforcing	16.23	35.2
3	Blood circulation-promoting and stasis-removing=>Heat-clearing and detoxifying	5.89	62.8
4	Heat-clearing and detoxifying =>Blood circulation-promoting and stasis-removing	5.89	12.8
5	Blood-stanching =>Heat-clearing and detoxifying	5.32	60.2
6	Heat-clearing and detoxifying =>Blood-stanching	5.32	11.5
7	blood circulation-promoting and stasis-removing=>Qi-reinforcing	5.02	53.5
8	Qi-reinforcing =>Blood circulation-promoting and stasis-removing	5.02	14.4
9	Qi-regulating =>Heat-clearing and detoxifying	4.78	58.2
10	Heat-clearing and detoxifying =>Qi-regulating	4.78	10.4

**Table 8:** Association rules of pharmacological mechanisms of modern medicines combined with CKI.

No.	Association rules	Support	Confidence
1	Immunomodulator =>Antineoplastic chemotherapeutic drugs	57.8	83.4
2	Antineoplastic chemotherapeutic drugs =>Immunomodulator	57.8	69.7
3	5-HT receptor antagonist drugs =>Antineoplastic chemotherapeutic drugs	48.1	96.6
4	Antineoplastic chemotherapeutic drugs =>5-HT receptor antagonist drugs	48.1	58.0
5	Antibiotics =>Antineoplastic chemotherapeutic drugs	40.3	80.0
6	Antineoplastic chemotherapeutic drugs =>Antibiotics	40.3	48.6
7	5-HT receptor antagonist drugs =>Immunomodulator	37.5	75.3
8	Immunomodulator =>5-HT receptor antagonist drugs	37.5	54.1
9	Antibiotics =>Immunomodulator	36.9	73.3
10	Immunomodulator =>Antibiotics	36.9	53.2

## Data Availability

The data that support the findings of this study are available from Institute of Basic Research in Clinical Medicine, China Academy of Chinese Medical Sciences, but restrictions apply to the availability of these data, which were used under license for the current study, and so are not publicly available. Data are however available from the authors upon reasonable request and with permission of Prof. Yanming Xie from Institute of Basic Research in Clinical Medicine, China Academy of Chinese Medical Sciences.

## Abbreviations

HIS: Hospital information system
RWS: Real-world study
EMR: Electrical medical record
CKI: Compound Kushen Injection
TCM: Traditional Chinese medicine
ARA: Association rule analysis.
